# Local Superimpositions Facilitate Morphometric Analysis of Complex Articulating Structures

**DOI:** 10.1093/icb/icab031

**Published:** 2021-04-27

**Authors:** Daniel Rhoda, Marion Segall, Olivier Larouche, Kory Evans, Kenneth D Angielczyk

**Affiliations:** Committee on Evolutionary Biology, University of Chicago, 5801 S Ellis Ave, Chicago, IL 60637, USA; Department of Herpetology, American Museum of Natural History, Central Park West at 79th Street, New York, NY 10024, USA; Department of BioSciences, Rice University, 6100 Main St, Houston, TX 77005, USA; Department of BioSciences, Rice University, 6100 Main St, Houston, TX 77005, USA; Negaunee Integrative Research Center, Field Museum of Natural History, 1400 South Lake Shore Drive, Chicago, IL 60605, USA

## Abstract

Articulating structures, such as the vertebrate skeleton or the segmented arthropod exoskeleton, comprise a majority of the morphological diversity across the eukaryotic tree of life. Quantifying the form of articulating structures is therefore imperative for a fuller understanding of the factors influencing biological form. A wealth of freely available 3D data capturing this morphological diversity is stored in online repositories such as Morphosource, but the geometric morphometric analysis of an articulating structure is impeded by arbitrary differences in the resting positions of its individual articulating elements. In complex articulating structures, where the angles between articulating elements cannot be standardized, landmarks on articulating elements must be Procrustes superimposed independently (locally) and then recombined to quantify variation in the entire articulating structure simultaneously. Here, we discuss recent advances in local superimposition techniques, namely the “matched local superimpositions” approach, which incorporates anatomically accurate relative sizes, positions, and orientations of locally-superimposed landmarks, enabling clearer biological interpretation. We also use simulations to evaluate the consequences of choice of superimposition approach. Our results show that local superimpositions will isolate shape variation within locally-superimposed landmark subsets by sacrificing size and positional variation. They may also create morphometric “modules” when there are none by increasing integration within the locally-superimposed subsets; however, this effect is no greater than the spurious between-module integration created when superimposing landmark subsets (i.e., articulating elements) together. Taken together, our results show that local superimposition techniques differ from conventional Procrustes superimpositions in predictable ways. Finally, we use empirical datasets of the skulls of wrasses and colubriform snakes to highlight the promise of local superimpositions and their utility. Complex articulating structures must be studied, and the only current solution to do so is local superimpositions.

## Introduction

**Fig. 1:**
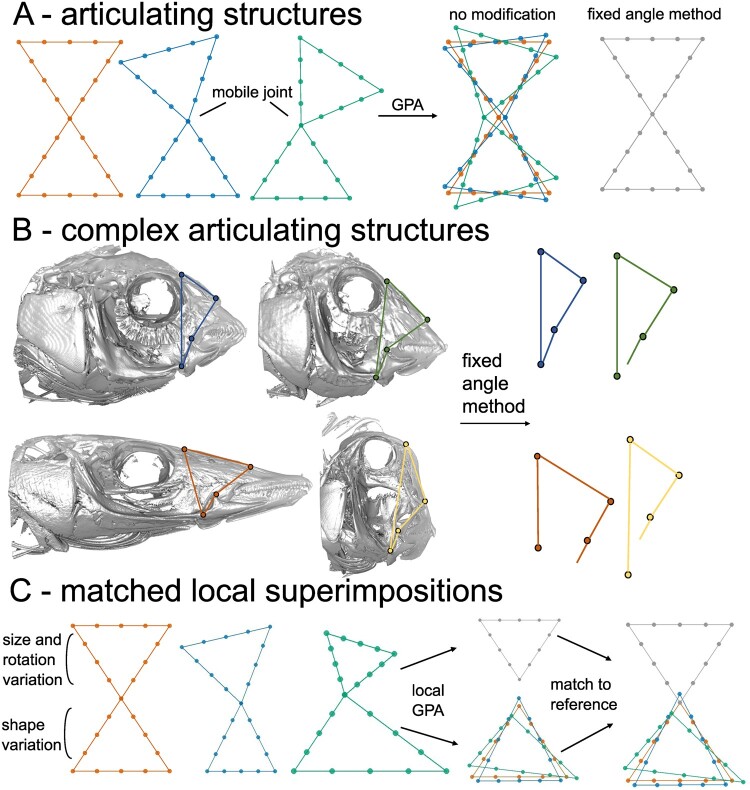
Articulating structures, with a mobile joint between articulating elements, should not be superimposed because arbitrary differences in articulation angle mask “real” landmark variation. (A) Three pairs of triangles, with no shape differences, have a mobile joint between the pairs causing their articulation angles to differ. A global superimposition with no modifications will create landmark variation across the entire structure when there is only variation in the articulation angle. The fixed angle method prevents this by standardizing the angle between all observations. (B) The fixed angle method is not applicable to complex articulating structures because the articulation angles are a function of the position of joints and relative sizes and positions of each articulating element. The 4-bar linkage systems in wrasses is used to show how each link cannot be rotated to a fixed angle while also preserving their natural articulations because these articulations are directly a product of each link’s relative size. (C) The matched local superimposition approach captures shape variation within each articulating element and places this variation at the relative position, orientation, and size of its corresponding element from a reference configuration. Each subset is individually superimposed with a GPA and “matched” onto their corresponding subset from a reference configuration. There is only rotational and size variation in the top triangles, and only shape variation in the bottom triangles. The matched local superimpositions procedure recovers the shape variation, or lack thereof, in each set landmark subset

Since the “revolution” in morphometrics, geometric morphometrics (GM) has provided organismal biologists with a powerful toolkit to visualize and quantify the link between morphological disparity and factors influencing biological form such as function, ecology, and development (Rohlf and Marcus 1993; Adams et al. 2004; Zelditch et al. 2004). Under the “Procrustes Paradigm” (Adams et al. 2013), Generalized Procrustes Analysis (GPA) is used to remove nonshape variation (position, orientation, and scale) from landmark data by scaling all configurations to unit centroid size, translating to a common location, and rotating to minimize the squared distance between each configuration and the mean shape (Gower 1975; Rohlf and Slice 1990). This process allows pure shape variation between landmark configurations to be quantified and compared to covariates of interest. GPA, however, is limited to rigid structures; in an articulating structure with a mobile joint, arbitrary differences in the resting position of separate articulating elements will confound the biologically relevant landmark variation of interest (Fig. 1A) (Adams 1999). Yet, kinetic articulating structures, such as the skulls of fishes and snakes, the limb segments of tetrapods, the body segments of arthropods, the component parts of flowers, even the entire vertebrate skeleton, comprise the majority of morphological diversity across the eukaryotic tree of life. Developing ways in which the form of articulating structures can be accurately quantified is imperative for a comprehensive understanding of the evolution of morphological disparity.

Analyzing shape variation in the individual articulating elements separately is one solution (i.e., statistically linking their morphologies to covariates of interest one-by-one), but it may be desirable to investigate shape variation of an articulating structure in a common coordinate space. Adams (1999) developed the “fixed angle method”, eloquently extended into three dimensions by Vidal-García et al. (2018) (Fig. 1A). In fixed angle methods, variation due to mobility (referred to as “preservational variation” hereafter) is erased by standardizing the angle between the landmarks of articulating elements for all observations, and then treating the modified landmarks as a rigid structure during GPA. This may be the best practice for GM analysis of simple articulating structures, like the cranium and mandible (Adams and Rohlf 2000; Adams 2004; Davis et al. 2016) or limb segments (Vidal-García and Keogh 2017), because it maintains the relative sizes of articulating elements while removing preservational variation. However, the fixed angle method is limited when considering complex articulating structures (Box 1), where individual rigid elements articulate with multiple other elements, possibly forming linkage systems or with numerous joints at different locations between observations. In these complex articulating structures, for example, the kinetic linkage system of a fish skull, individual articulating elements cannot be rotated to fixed angles while also preserving their articulations because the relative size of each rigid element determines its resting articulation angle with the others (Fig. 1b).

Superimposing articulating elements separately (i.e., “local” superimpositions), scaling each separate fit to their relative sizes (i.e., the subset’s centroid size divided by the total centroid size of all subsets, possibly incorporating *a priori* user-defined weights for each subset), and then concatenating the landmarks into the same coordinate space are a convenient and simple approach to analyze a complete articulating system simultaneously, no matter the complexity (Collyer et al. 2020). We refer to this procedure as the “combined subsets” approach. In this procedure, each landmark subset is rotated to its principal axis and translated to the origin after scaling, and then treated as an already-superimposed rigid structure in subsequent analyses. Hellert (2019) analyzed the separate limb elements of birds in a common coordinate space by superimposing each element separately and then concatenating the separate superimpositions. However, unlike the combined subsets approach, the individual superimpositions were set apart along the *X*-axis so that each limb element was located in a different position. This enabled within- and between-module landmark covariance to be considered at once while also giving clarity during the visualization of results from exploratory analyses of modularity. Rhoda et al. (2021) presented another local superimposition approach, where an anatomically accurate reference landmark configuration was used as an anchor to place locally-superimposed landmark subsets onto their corresponding subset in the reference configuration (Fig. 1c). This “matched local superimpositions” procedure is similar to the combined subsets approach but differs in that it places locally-superimposed landmark subsets in a position and orientation reflecting an anatomically accurate reference configuration rather than at the origin of the coordinate system and aligned to their respective principal axes.

These procedures for quantifying the shapes of articulating structures have different strengths and weaknesses, and each may be the best practice depending on the system or question of interest. Local superimposition methods differ from the fixed angle method in the number of superimpositions that are performed, which is significant because GPA homogenously redistributes landmark variance across a landmark configuration even when there is none (Rohlf and Slice 1990). For example, consider an ontogenetic sequence of a skull displaying positive allometry in the length of the face but isometry in the braincase. In this scenario, there is no “real” shape variation in the braincase, but because there is in the face the centroid positions will shift anteriorly through ontogeny, causing the braincases of older skulls to be disproportionately smaller and shifted posteriorly following a GPA. Such redistribution of variance is a particularly important concern in the study of morphological integration and modularity (i.e., correlations between anatomical traits and their assembly into semi-independently varying clusters of traits, respectively, Olson and Miller 1958; Wagner 1996) because it may dampen a modular signal. Cardini (2019) demonstrated that a global Procrustes superimposition produces covariation between landmark subsets when there is only isotropic variation, causing statistical tests of morphological integration to erroneously recover statistically significant integration between the subsets. Locally superimposing the modules eliminated this effect, but the covariance structure is still altered within each locally-superimposed subset, and the extent to which local superimpositions promote modularity is less understood (Cardini 2019).

Interrogating the evolution of form in complex articulating structures is necessary because of the vast morphological diversity they represent, yet the best way to analyze such structures has received little attention. Here, we use simulations to consider the strengths and weaknesses of local superimposition procedures and highlight their utility with empirical datasets of highly kinetic snake and wrasse skulls. We also present preliminary analyses of the effect of local superimpositions on the covariance structure of a GM dataset and evaluate its implications for studies of morphological integration and modularity.

## Local and global superimpositions differ in predictable ways

When an anatomical structure is composed of component parts that are not spatially fixed, and either do not articulate or form a complex articulating structure, locally superimposing each component and concatenating the shape variables is the only current solution to consider the shapes of each component simultaneously during analysis.

The matched local superimpositions procedure incorporates information about the relative orientation, position, and scale of an anatomically realistic reference landmark configuration (Fig. 1C) (Rhoda et al. 2021). This procedure is a direct extension of that advocated by Collyer et al. (2020) with relative orientation and position of each subset included, permitting clearer biological interpretation of patterns of shape variation. R code for this procedure is attached in the Supplementary data. The procedure first scales the reference configuration to unit centroid size, translates to the origin, and calculates the centroid sizes and centroid positions of each landmark subset defined by the input partition (effectively retaining the proportional sizes of subsets as in the combined subsets approach if each subset weight = 1). Landmark subsets should represent the individual rigid articulating elements of an articulating structure (or the smallest potential module, as discussed below). Each landmark subset is locally superimposed using GPA, then scaled to the centroid size, and translated to the centroid position of its corresponding subset from the (scaled and translated) reference configuration. The locally-superimposed configurations are then rotated to minimize the squared distance between the reference subset and the mean shape of the locally-superimposed subset. Because each observation is scaled, translated, and rotated by the same amount, pure shape variation within each subset is preserved throughout the procedure.

The matched local superimposition procedure extracts the pure shape variation within each landmark subset and then places this variation at the position, orientation, and scale defined by a reference configuration. This procedure is applicable to any GM dataset as long as an anatomically-realistic reference landmark configuration can be obtained. The reference landmark configuration may be the mean configuration of a global superimposition of the raw landmark data if the raw landmark data are in repeatable positions (with preservational variation minimized, meaning that the mean configuration will resemble the repeatable position). Alternatively, the reference configuration may be from a single specimen, for example, in its CT-scanned position. The choice of reference configuration is consequential because the relative sizes of its subsets determine the “importance” of each subset during analysis (i.e., a larger sized subset will account for proportionally more total shape variation), so the reference configuration should be as anatomically realistic as possible (Supplementary Fig. S1). When dealing with articulating structures, no “true” position exists because there is a mobile joint or joints (e.g., there is no single “true” angle at which elements of a tetrapod limb articulate, but many “natural” angles may exist; Collyer et al. 2020), and thus, the fixed angle or matched local superimposition procedures necessarily incorporate semi-arbitrary choices on the relative orientations of landmark subsets. Additionally, by anchoring each subset to an anatomically realistic reference configuration, the matched local superimpositions procedure allows users to visualize shape variation with each element in a homologous position and orientation, facilitating biological interpretation. The landmark subsets may overlap at times (especially in particularly variable datasets) and will only articulate perfectly in the reference configuration, because their positions and orientations are fixed but shapes vary (see the bottom triangle in the product of the matched local superimpositions in Fig. 1C).

### Simulation I

**Fig. 2:**
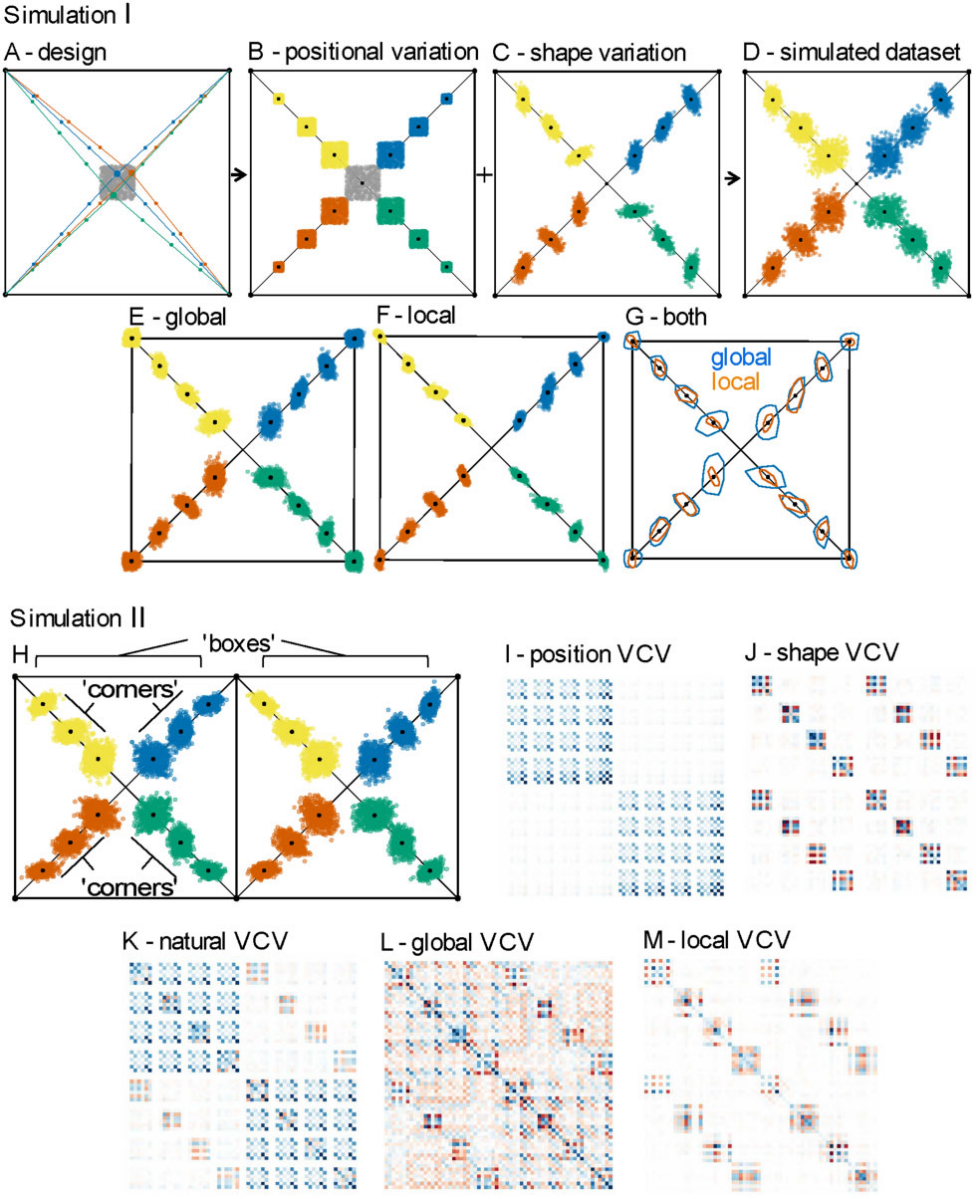
Simulating positional and shape variation in a complex articulating structure demonstrates that global superimpositions maintain positional variation, whereas local superimpositions isolate pure shape variation within each locally-superimposed landmark subset. Simulation I: (A) Midpoints (in gray), representing the joint between four “articulating elements,” were randomly simulated in a restricted location within a square, simulating positional variation (B). (C) Correlated shape variation (stronger covariation within each articulating element, represented by color) was introduced to create the full simulated dataset (D), and a (E) global and (F) matched local superimpositions were then applied to visualize the patterns and magnitudes of landmark variation each superimposition procedure captures. Simulation II: (H) The simulation was repeated with two adjacent squares, with correlated shape variation introduced between squares, represented by color. The “boxes” and “corners” labels denote the competing hypotheses of modularity. Each column and row in the VCV matrices represent a shape variable (i.e., the *x* or *y* value of a landmark). Lighter colors of cells represent values closer to zero and darker colors (red or blue) represent values farther away from one (i.e., stronger (co)variance between variables). Visualization of the VCV matrices reveals that local superimpositions capture the introduced shape variation and that the global superimposition maintains the positional variation but spreads it across the entire landmark configuration. (I) VCV matrix of just the positional variation created by randomly sampling midpoints. The two blue squares in the VCV matrix correspond to the separate boxes. (J) VCV matrix of just the simulated shape variation. (K) VCV matrix of the full simulated dataset before any superimposition. (L) VCV matrix after a global superimposition. (M) VCV matrix after matched local superimpositions

Local superimposition procedures will sacrifice the positional and relative size variations between observations in favor of pure shape variation (Baab 2013). We demonstrate this point by performing simulations on an artificial linkage system: a square with 4 sets of equally-spaced landmarks connecting each vertex to a point within the square. Inside of this square, we randomly sampled 500 “midpoints” within a restricted location. For each replicate, we placed 3 equally-spaced landmarks between each vertex and the midpoint. By randomly sampling midpoints before calculating equally-spaced landmarks, the relative sizes and positions of each set of equally-spaced landmarks for each replicate depend on the midpoint location (Fig. 2A, B), just as how the resting articulation angles in the 4-bar linkage systems of wrasse depend on the relative sizes and positions of its constituent bones (Fig. 1B). In this analogy, the sets of equally-spaced landmarks represent articulating elements, each connected to a common joint represented by the midpoint. This design is meant to simulate the relative size and positional variation that exists in a complex articulating structure completely independent of shape or preservational variation. We used the “mvrnorm” function in the R package *MASS* (Ripley et al. 2013) to introduce simulated shape variation, drawn from a normal distribution, with a variance–covariance (VCV) matrix constructed that induced covariation within the sets of equally-spaced landmarks but not between (Fig. 2C). We then rotated the direction of variation within each landmark by a random angle because the “mvrnorm” function induces covariation in a common direction, which would immediately be removed during the translation step of a GPA. Neither positional nor shape variation was added onto any of the 4-vertex landmarks so that the full simulated dataset represented a “natural” superimposition from which we could observe the true patterns of landmark covariation before any superimpositions (Fig. 2D; Goswami et al. 2019). Finally, we applied a global superimposition and matched local superimpositions to the full simulated dataset with the midpoints removed, each set of equally-spaced landmarks as subsets, and the mean configuration from the global superimposition as reference. The matched local superimpositions should extract only the simulated shape variation, whereas the global superimposition should preserve the simulated positional and size variation introduced by modifying the midpoint location.

The global superimposition preserves the simulated positional variation but distributes much of it onto the 4 vertex landmarks, which each have rectangular landmark scatters (Fig. 2E). These rectangular scatters are created because the centroids of each configuration change as a function of the simulated positional variation more than they do because of shape variation, such that during the translation step of the global superimposition the entire configurations will be translated predominantly based on each replicate’s random midpoint location. Conversely, as predicted, the matched local superimpositions better reflect the shape variation (Fig. 2F). The directions of shape (co)variation are affected by both superimpositions, but especially by the local superimpositions. The local superimpositions distribute variance within each subset rather than the whole configuration, shown by the ellipsoidal scatters around the vertex landmarks. Because covariation was introduced within each subset, centroid positions vary slightly between observations in a consistent way, creating the ellipsoidal scatters in the vertices via the same mechanism by which the global superimposition creates rectangular scatters. Another key difference shown in these simulations is that local superimpositions will have a lower absolute variance because each local superimposition incorporates fewer landmarks than a global superimposition, resulting in tighter fits than if all landmarks were considered (Fig. 2G) (Collyer et al. 2020).

### Simulation II

To statistically analyze the effect that global versus local superimpositions have on the inference of patterns of positional and size variation versus shape variation, we conducted a second version of the “square” simulation. This version used 2 squares adjacent to one another and simulated covariation within the equally-spaced landmarks from the same vertices and between the equally-spaced landmarks from similar corners of the different boxes (Fig. 2H). We visualized the VCV matrices of the simulated data at each step of the experiment and tested 2 *a priori* hypotheses of modularity on both the globally and locally-superimposed data (Fig. 2I–M). We measured modularity using the Covariance Ratio (CR) in *geomorph*, which is a ratio of between-module integration to within-module integration, and its effect size (Z_CR_), where lower values reflect stronger modular signals (Adams and Otárola-Castillo 2013; Adams 2016; Adams and Collyer 2019). The “boxes” hypothesis of modularity proposed the 4 sets of equally-spaced landmarks from the different boxes as 2 separate modules and the “corners” hypothesis proposed that the equally-spaced landmarks from the same corner of the separate boxes were modules (4 modules). The “boxes” hypothesis should be more supported after a global superimposition because it preserves relative size and positional variation, whereas the “corners” hypothesis should gain more support in the matched local superimpositions because it favors within-module pure shape variation.

As predicted, the “boxes” hypothesis is supported in the global superimposition (CR = 0.861, *P* = 0.004, Z_CR_ = –2.444) but not the local superimposition dataset (CR = 0.9616, *P* = 0.274, Z_CR_ = –0.648). Both superimposition procedures showed support for the “corners” hypothesis, but the local superimpositions procedure recovered a much lower CR value than the global superimpositions despite their having similar effect sizes (global: CR = 0.625, *P* = 0.001, Z_CR_ = –5.494; local: CR = 0.064, *P* = 0.001, Z_CR_ = –6.15). This is because there is a lower absolute variance in the local superimpositions, as discussed above, indicating that Z_CR_ values should not be directly compared between superimposition procedures. However, the relative order of support of competing hypotheses can be compared between different procedures. This point is especially important because it means that locally-superimposed subsets should represent the smallest possible modules, because modules would be both locally and globally superimposed if not. Visual inspection of the VCV matrices further demonstrates the effect that the choice of superimposition can have on the covariance structure of a GM dataset (Fig. 2I–M). The local superimposition VCV matrix is nearly identical to the shape only VCV matrix, differing only in that the vertex landmarks now covary with their corresponding equally-spaced landmarks. The global superimposition VCV matrix differs dramatically from the full simulated VCV as well as both position- and shape-only VCV matrices. The shape covariation is subtly visible in the global VCV matrix but is overshadowed by the homogenously redistributed variance. Altogether, these simulations validate our expectations and illustrate the primary differences between global versus local superimpositions.

## The utility of local superimpositions, examples from wrasses and snakes

**Fig. 3:**
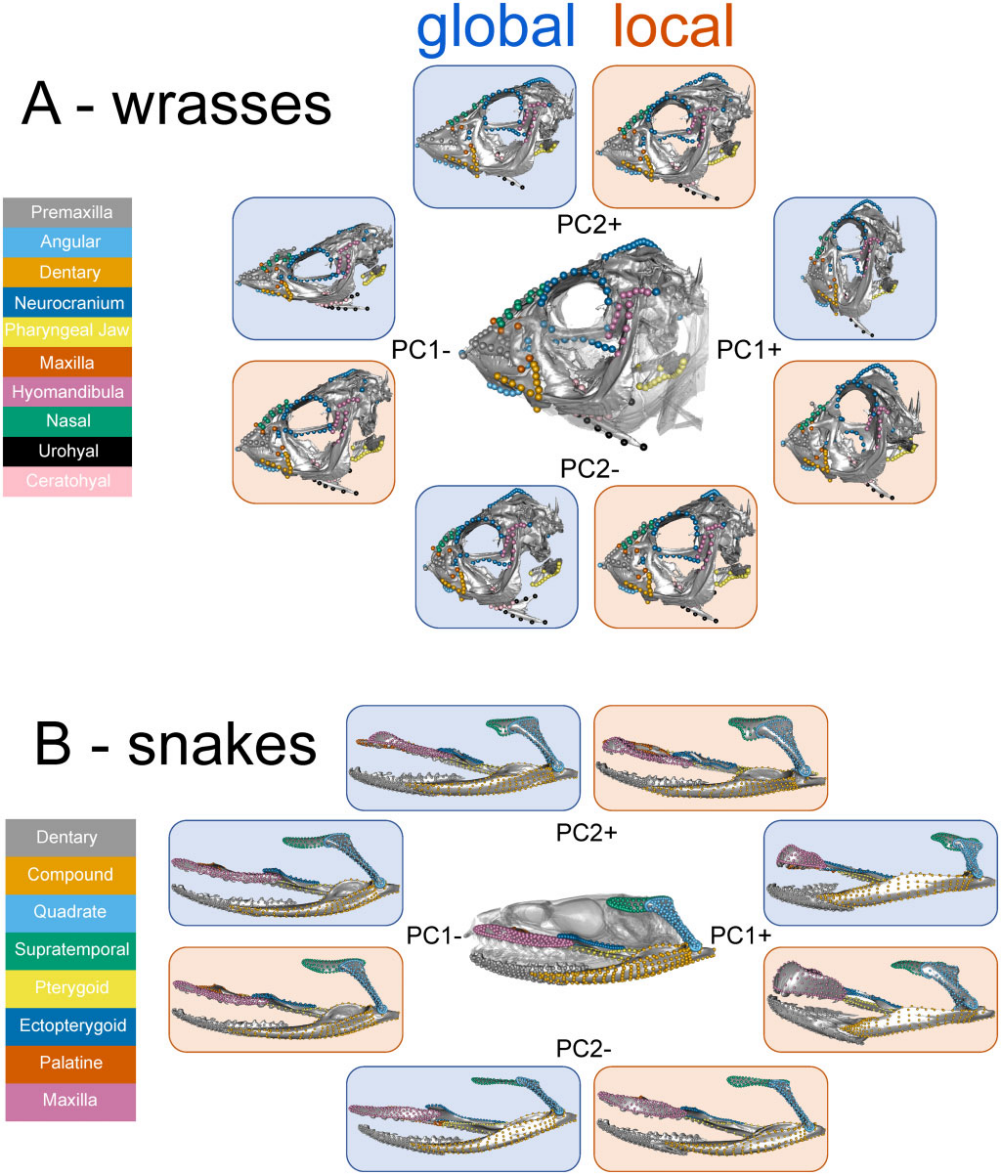
Patterns of landmark variation along principal components 1 and 2 of the local and global superimpositions in (A) wrasses and (B) colubriform snakes. Landmark configurations represent the extremes of the PC axes with a mesh warped to its shape. Configurations from the global superimposition are on blue backgrounds and local superimposition configurations are on orange backgrounds

We present 2 empirical examples to demonstrate the promise of local superimpositions in analyzing complex articulating structures, the jaws of aquatic-foraging snakes (colubriform species from Rhoda et al. 2021; non-colubriforms were excluded because of significant structural changes that are beyond the scope of this study; Cundall and Irish 2008), and the skulls of wrasses (Evans et al. 2019). The snake dataset contains 1335 total landmarks and 29 species, and the wrasse dataset contains 178 landmarks and 30 species. Semi-landmarks in both datasets were slid by minimizing bending energy (Gunz et al. 2005). Both represent complex articulating structures unsuitable for the fixed angle method because the bones articulate to form linkage systems where natural articulation angles depend on the relative sizes and positions of the constituent bones. The preservational variation is small in comparison to the interspecific positional and rotational variation captured in the datasets; the bones still articulate, there are many soft tissue connections reducing preservational variation, and each specimen in both datasets was in a neutral resting position by ensuring the mouths were closed with no visible deformation in the overall configuration of the skull prior to landmark placement. Because both datasets contain morphologically variable species arguably in homologous positions, the major axes of shape variation after a global superimposition should still reflect the prominent patterns of interspecific shape variation within the datasets. Although these axes may not be exactly the same as if there was no preservational variation, if the biologically noteworthy variation is greater than this preservational variation, the global superimposition can be compared with a local superimposition procedure to better understand how the exclusion of relative size and positional variation affects results in an empirical dataset. We globally superimposed each dataset as if it was a rigid structure and then locally superimposed it using the matched local superimposition approach with the mean configuration from the global superimposition as the reference. We visualized the major axes of shape variation using principal components analysis (Fig. 3). To mathematically compare how species are distributed throughout morphospace of the alternative superimposition methods, Procrustes distance (dissimilarity) matrices were calculated for each superimposition method and compared with a Mantel test (Smouse et al. 1986) for both datasets. To determine how these different superimpositions affect the inference of modularity in empirical datasets, we directly compared alternative hypotheses of modularity that would be expected in certain functional or developmental contexts using the CR in a phylogenetic framework (Pyron and Burbrink 2014; Adams 2016; Aiello et al. 2017; Adams and Collyer 2019; Supplementary Fig. 2).

As expected, the major axes of shape variation in the global superimposition recovered variation in the relative sizes and positions of the component parts of the skulls, whereas the local superimpositions emphasized pure shape variation within each part (Fig. 3). In the wrasse dataset, PC1 captures 41.1% of the variance, with narrow and elongated skulls at the negative side of the axis and shorter, dorso-ventrally inflated skulls at the positive end (Fig. 3A). The orientation of the mouth (maxilla, premaxilla, dentary, angular) and position of the pharyngeal jaw varies along PC2 (15% of shape variation). In the wrasse local superimpositions dataset, dorso-ventral elongation of the neurocranium drives shape variation along PC1 (34.7% of variation), while the length of the supraoccipital crest varies along PC2 (14.3% of variation) (Fig. 3A).

A more posteriorly rotated quadrate, slender maxilla, and dorsally concaved mandible are observed at the negative end of PC1 (40.7% of variation) in the snake global superimposition dataset. Along PC2 (14% of variation) the latero-medial orientation of the mandible and maxilla varies, as well as the slenderness of the supratemporal (Fig. 3B). Besides posterior rotation of the quadrate in PC1 (34% of variation) and orientation of the mandible and maxilla in PC2 (13.7% of variation), the patterns of shape variation in the local superimpositions dataset mimic those that are found in the global superimpositions (Fig. 3B).

In both the wrasse and snake datasets, species are distributed along PC1 in similar ways regardless of superimposition type; this is also true for PC2 in the snake dataset (Supplementary Figs. S3–S6). Pure shape variation within landmark subsets is intimately related to the positional variation captured in the global superimpositions. Wrasse skulls at PC1+ would not be elongated dorsoventrally if not for the depth of the neurocranium, and the snake quadrates would not be posteriorly rotated at PC1− if they were not slender and elongated. This does not mean that pure shape and positional variation are always related, but in an empirical dataset, it is likely that the factors driving positional variation are also imprinting as pure shape variation, allowing these factors to be linked to morphology during the analysis of a local superimposition dataset. This is corroborated by the Mantel test results, which showed high correlations between superimposition methods (snakes: *r* = 0.853, wrasses: *r* = 0.847), indicating that species are distributed across the morphospaces in similar ways.

The results from the modularity analyses support this assertion. In the wrasse dataset, the different superimposition procedures recovered the greatest support for the same hypothesis of modularity, and in the snake dataset, similar relative support for alternative hypotheses was found as well (Tables 1 and 2). The hypothesis of complete modularity (all landmark subsets as separate modules) was most supported in the wrasse dataset and the 2-module hypothesis of the mandible, quadrate, and maxilla as a module separate from the rest of the configuration was most supported in the snake dataset. In the snake dataset, the 2-module hypothesis of the mandible, quadrate, and maxilla separate from the rest of the configuration received the least amount of support in the global superimposition dataset but was the most supported in the matched local superimpositions. This is likely because the position and orientation of the maxilla is in part dependent on that of the rest of the upper jaw through its medial joints with the palatine and ectopterygoid (Fig. 3B), but its shape variation is co-adapted with the mandible to accommodate certain prey items and foraging strategies (Savitzky 1983). In this case, the global superimposition recovers positional variation and the local superimpositions recovers only shape variation, consistent with expectations from the square simulations (Fig. 2). Interestingly, in the snake dataset, the CR values of each hypothesis are larger in the global superimposition dataset, whereas in the wrasse dataset, the CR values are smaller in the global superimposition dataset (besides the hypothesis of complete modularity). This result indicates that even if local superimpositions promote modularity or global superimpositions promote integration (Cardini 2019, and further discussed below), in an empirical dataset these effects do not necessarily overshadow the potentially different “true” patterns of covariance in positional versus pure shape variation. These examples show that in an empirical dataset, results from local superimpositions will differ from global superimpositions in predictable ways, concordant with the box simulations.

**Table 1:** Results of modularity analyses of the wrasse dataset

Wrasse dataset	Global			Local		
Hypothesis	CR	*P*-value	Z_CR_	CR	*P*-value	Z_CR_
[premaxilla, angular, dentary, pharyngeal jaw, maxilla] + other	0.901	0.001	–7.328	0.955	0.039	–2.139
[nasals, neurocranium] + other	0.864	0.001	–8.897	0.963	0.062	–1.722
[nasals, neurocranium] + [premaxila, angular, dentary, maxilla] + other	0.826	0.001	–10.431	0.875	0.001	–5.958
Complete modularity	0.757	0.001	–10.759	0.653	0.001	–9.661

Darker green cells denote lower effect sizes and a stronger modular signal. “Other” refers to a module of all bones not mentioned

**Table 2:** Results of modularity analyses of the colubriform snake dataset

Colubriform snake dataset	Global			Local		
Hypothesis	CR	*P*-value	Z_CR_	CR	*P*-value	Z_CR_
mandible + other	0.828	0.001	–28.172	0.626	0.001	–20.627
[mandible, quadrate, supratemporal] + other	0.691	0.001	–30.371	0.680	0.001	–22.937
[mandible, quadrate, maxilla] + other	0.895	0.001	–27.127	0.672	0.001	–23.056
[mandible] + [quadrate, supratemporal] + other	0.695	0.001	–29.992	0.649	0.001	–21.719
[mandible, quadrate, supratemporal] + maxilla + other	0.671	0.001	–29.510	0.639	0.001	–20.266
[dentary, maxilla] + [compound, quadrate, supratemporal] + other	0.663	0.001	–30.188	0.620	0.001	–22.699
[mandible] + [quadrate, supratemporal] + [ectopterygoid, pterygoid, palatine] + maxilla	0.658	0.001	–29.909	0.616	0.001	–21.402
Complete modularity	0.571	0.001	–28.683	0.531	0.001	–18.557

The dentary and compound bones are referred collectively as the mandible. “Other” refers to a module of all bones not mentioned

## All superimpositions modify covariance structure

Complex articulating structures are excellent systems for the study of morphological integration and modularity because they are composed of articulating parts that may have distinct developmental origins and that may work in concert to fulfill their functions (Hallgrímsson et al. 2009). Local superimpositions allow us to place the shape variations of these component parts in a common coordinate space, facilitating the simultaneous analysis of within- and between-module covariance. Understanding how local superimpositions disrupt the statistical inference of modularity is therefore an important consideration. Global superimpositions can create covariation between landmark subsets (i.e., modules, articulating elements) when there is only random isotropic variation, leading to spurious results in integration tests (Cardini 2019). Locally superimposing each module removes this erroneous between-module integration, but the integration-inducing aspects of GPA that cause erroneous results in the global superimposition persist within each locally-superimposed landmark subset. Local superimpositions may promote modularity either by increasing within-module covariance or reducing between-module covariance associated with size (Cardini 2019).

To investigate how the choice of superimposition influences tests of modularity, we simulated both isotropic noise and modular variation on two landmark configurations: (1) a set of 6 triangles, each representing a module and (2) the Type I landmarks from the mean shape of the snake dataset with each articulating element as a module (Tables 3 and 4). For each module, simulated covariance was greater within the subset than between it and other subsets, and within-landmark variation for each landmark was larger than covariation with any other landmark. We applied a global superimposition, matched local superimpositions, and local superimpositions using the “combine.subsets” function in *geomorph* (with the “weights” and “CS.sets” parameters set to NULL so that the relative sizes from the input subsets were preserved, as in the matched local superimpositions) to both datasets for both models of landmark covariance. For all datasets, we tested the *a priori* hypothesis of each landmark subset as a separate module using the CR. We used 2-block partial least-square analyses to test the magnitude of between-module integration (the averaged effect size, _Z_PLS, between each module, Rohlf and Corti 2000; Adams and Collyer 2016), and relative eigenvalue variance (Pavlicev et al. 2009; Machado et al. 2019) to assess the magnitude of within-module integration. The less a superimposition procedure alters the covariance structure of a dataset, the more its results should match results from the natural superimposition. We used Mantel tests to statistically compare the Procrustes distance matrices of the global and matched local superimpositions of each simulated dataset. We found that in both datasets when isotropic noise is simulated, a strong modular signal is recovered in both local superimposition procedures (Table 3). A weak but significant modular signal is recovered in the global superimposition of the triangle dataset. When landmark variation is simulated, the centroid positions and sizes of each subset will change slightly, even when randomly rotating the directions of covariance. During local superimpositions of these subsets, each observation will be scaled and translated to a common size and position, creating covariation within the subsets but not between because each subset is superimposed separately (Goswami et al. 2019). This is supported by the eigenvalue variance results, which showed that the average overall magnitude of integration within each module is greater in the local superimposition datasets than in the natural or global superimposition datasets (Tables 3 and 4). In the absence of any covariation, this creates a strong modular signal, but it is important to note that this is an unrealistic expectation for empirical datasets because morphological integration is ubiquitous in biological systems (Wagner et al. 2007). Our isotropic noise simulations are only used to demonstrate that the integration-inducing aspects of GPA highlighted by Cardini (2019) may promote modularity when using local superimpositions by increasing within-module integration. Along these lines, by homogenously redistributing variance across entire landmark configurations, global superimpositions inflated the degree of between-module integration in both datasets and models of variation, corroborating these troubling findings (_Z_PLS values, Tables 3 and 4). All superimposition procedures maintained the simulated modular variation (Table 4), with all Z_CR_ values being similar to the natural superimposition effect sizes. The matched local superimpositions procedure produced CR values nearly identical to the natural superimposition values, and the global and combined subset approaches recovered marginally larger CR values, suggesting greater between-module covariation.

**Table 3:** Integration test results from the isotropic noise simulations

Isotropic noise										
Datasets	Triangles					Snakes				
	CR	*P*-value	Z_CR_	Mean zPLS	REV	CR	*P*-value	Z_CR_	Mean zPLS	REV
Natural superimposition	1.007	0.144	–1.068	–2.295	0.049	1.069	0.883	1.174	0.867	0.05
Global superimposition	0.971	0.003	–2.999	3.043	0.053	1.042	0.396	–0.223	2.056	0.056
Matched local superimpositions	0.444	0.001	–13.883	–0.719	0.105	0.296	0.001	–7.543	1.243	0.165
Combined subsets	0.512	0.001	–14.845	0.157	0.093	0.340	0.001	–6.275	1.448	0.15

Note that a higher relative eigenvalue variance value corresponds to a higher degree of morphological integration. Different colors correspond to the different landmark subsets that are locally superimposed in the matched local superimpositions and combined subsets approaches, as well as the patterns of simulated modular variation. REV: relative eigenvalue variance

**Table 4:** Integration test results from the modular variation simulations

Modular variation										
Datasets	Triangles					Snakes				
	CR	*P-*value	Z_CR_	Mean zPLS	REV	CR	*P*-value	Z_CR_	Mean zPLS	REV
Natural superimposition	0.067	0.001	–11.408	–1.29	0.676	0.0653	0.001	–9.194	0.28	0.637
Global superimposition	0.102	0.001	–11.088	2.59	0.668	0.121	0.001	–8.355	2.657	0.596
Matched local superimpositions	0.064	0.001	–10.606	–0.21	0.67	0.070	0.001	–10.285	–0.248	0.516
Combined subsets	0.105	0.001	–10.544	–1.12	0.695	0.082	0.001	–11.014	–0.373	0.506

Note that a higher relative eigenvalue variance value corresponds to a higher degree of morphological integration. Different colors correspond to the different landmark subsets that are locally superimposed in the matched local superimpositions and combined subsets approaches, as well as the patterns of simulated modular variation. REV: relative eigenvalue variance

Despite these discrepancies in covariance structure, results from Mantel tests showed that the global and matched local superimpositions Procrustes distance matrices were all highly correlated (triangle isotropic: *r* = 0.928, snake isotropic: *r* = 0.843, triangle modular: *r* = 0.993, snake modular: *r* = 0.966), meaning that the distances between species in morphospace are similar between the two superimposition methods.

Taken together, these preliminary findings suggest that all three superimposition procedures tested here may lead to the recovery of erroneous patterns of integration. The local superimposition procedures both promote modularity when there is only isotropic noise because they increase within-module integration (without necessarily increasing between-module integration) in the same way that global superimpositions create whole-configuration integration when there is none. The global superimpositions increase between-module integration both under isotropic and modular variations. Importantly, the isotropic variation modeled here is not realistic, so the extent to which local superimpositions promote modularity when faced with heteroscedastic or completely integrated variation is an area for future research.

## Conclusions

Local superimpositions are essential for the morphometric analysis of complex articulating structures. These systems range from the linkage systems composing the hyperkinetic skulls of snakes or fishes as presented here, to the carpals and tarsals of tetrapod appendages, to the feeding apparatuses of insects, to a myriad of other biological systems. The promise of local superimpositions is particularly exciting as it may be the only means for whole-body GM analysis in a common coordinate space for some organisms, for instance, in the skeletons of felids (Randau and Goswami 2018) or birds (Bjarnason and Benson 2021). Further, leveraging the wealth of freely available 3D data within online repositories such as Morphosource, Phenome10k, MorphoMuseuM, and others (Boyer et al. 2016; Lebrun and Orliac 2016) will expedite comparative studies of phenotypic evolution in complex articulating structures.

There are key differences between local and global superimpositions. Local superimpositions will have a lower absolute variance and only consider pure shape variation within subsets, whereas global superimpositions maintain the relative size and positional variation between subsets. Importantly, as demonstrated using simulations, these differences are predictable, and although it is true that positional and relative size variation is lost in a local superimposition approach, the incorporation of this information during a global superimposition actually confounds the pure shape variation within potential modules. Because relative size and positional variation between modules is maintained, analyses of modularity within a global superimposition are analyzing covariation in form, not pure shape. Thus, when analyzing a modular rigid structure, these different approaches may be used jointly to better understand how the inclusion of positional and size variation between modules or the emphasis of pure shape affects the inference of modularity and the processes that generate these patterns. Unlike rotation and position, relative size variation of landmark subsets between observations is independent of preservational variation. Development of local superimposition procedures that maintain this size variation (as fixed angle methods do) while also fixing angles between component parts of a complex articulating structure (e.g., “iterative rearticulations” and “superimposed centroids” approaches in Rhoda et al. 2021) should be an avenue for future research.

Local superimpositions may induce a modular signal when there is none, but this is likely less of a concern in empirical datasets with anisotropic patterns of variation. Moreover, this concern does not appear to be any more severe than how global superimpositions promote between-module integration when there is none, as both of these modifications to the covariance structure spring from the same processes inherent to any GPA. Further development of superimposition methods assuming more biologically realistic models of covariance will alleviate these concerns.

Ultimately, any quantitative analysis of morphological integration is an imperfect estimation of the processes that produce relationships among traits; local superimpositions facilitate these analyses in kinetic articulating structures with predictable consequences. Taken together, these findings demonstrate that local superimpositions enable the morphometric analysis of complex articulating structures.

## Supplementary Material

icab031_Supplementary_Data

## References

[icab031-B1] Adams DC. 1999. Methods for shape analysis of landmark data from articulated structures. Evol Ecol Res 1:959–70.

[icab031-B2] Adams DC, Rohlf FJ. 2000. Ecological character displacement in Plethodon: biomechanical differences found from a geometric morphometric study. Proc Natl Acad Sci 97:4106–11. 10760280 10.1073/pnas.97.8.4106 PMC18164

[icab031-B3] Adams DC. 2004. Character displacement via aggressive interference in Appalachian salamanders. Ecology 85:2664–70.

[icab031-B4] Adams DC, Rohlf FJ, Slice DE. 2004. Geometric morphometrics: ten years of progress following the ‘revolution’. Ital J Zool 71:5–16.

[icab031-B5] Adams DC, Rohlf FJ, Slice DE. 2013. A field comes of age: geometric morphometrics in the 21st century. Hystrix 24:7.

[icab031-B6] Adams DC, Otárola‐Castillo E. 2013. geomorph: an R package for the collection and analysis of geometric morphometric shape data. Methods Ecol Evol 4:393–9.

[icab031-B7] Adams DC, Collyer ML. 2016. On the comparison of the strength of morphological integration across morphometric datasets. Evolution 70:2623–31. 27592864 10.1111/evo.13045

[icab031-B8] Adams DC. 2016. Evaluating modularity in morphometric data: challenges with the RV coefficient and a new test measure. Methods Ecol Evol 7:565–72.

[icab031-B9] Adams DC, Collyer ML. 2019. Comparing the strength of modular signal, and evaluating alternative modular hypotheses, using covariance ratio effect sizes with morphometric data. Evolution 73:2352–67. 31657008 10.1111/evo.13867

[icab031-B10] Aiello BR, Westneat MW, Hale ME. 2017. Mechanosensation is evolutionarily tuned to locomotor mechanics. Proc Natl Acad Sci USA 114:4459–64. 28396411 10.1073/pnas.1616839114 PMC5410822

[icab031-B7563482] Baab KL. 2013. The impact of superimposition choice in geometric morphometric approaches to morphological integration. J Hum Evol 65:689–92. 23954167 10.1016/j.jhevol.2013.07.004

[icab031-B11] Bjarnason A, Benson RBJ. 2021. A 3D geometric morphometric dataset quantifying skeletal variation in birds. MorphoMuseuM 7:e125.

[icab031-B12] Boyer DM, Gunnell GF, Kaufman S, McGeary TM. 2016. Morphosource: archiving and sharing 3-D digital specimen data. Paleontol Soc Pap 22:157–81.

[icab031-B13] Cardini A. 2019. Integration and modularity in Procrustes shape data: is there a risk of spurious results? Evol Biol 46:90–105.

[icab031-B14] Collyer ML, Davis MA, Adams DC. 2020. Making heads or tails of combined landmark configurations in geometric morphometric data. Evol Biol 47:193–205.

[icab031-B15] Cundall D, Irish F. 2008. The snake skull. Biol Reptilia 20:349–692.

[icab031-B16] Davis MA, Douglas MR, Collyer ML, Douglas ME. 2016. Deconstructing a species-complex: geometric morphometric and molecular analyses define species in the Western Rattlesnake (Crotalus viridis). PloS One 11:e0146166. 26816132 10.1371/journal.pone.0146166 PMC4731396

[icab031-B17] Evans KM, Williams KL, Westneat MW. 2019. Do coral reefs promote morphological diversification? Exploration of habitat effects on labrid pharyngeal jaw evolution in the era of big data. Integr Compar Biol 59:696–704. 10.1093/icb/icz103 31199432

[icab031-B18] Goswami A, Watanabe A, Felice RN, Bardua C, Fabre AC, Polly PD. 2019. High-density morphometric analysis of shape and integration: the good, the bad, and the not-really-a-problem. Integr Compar Biol 59:669–83. 10.1093/icb/icz120 PMC6754122 31243431

[icab031-B19] Gower JC. 1975. Generalized procrustes analysis. Psychometrika 40:33–51.

[icab031-B20] Gunz P, Mitteroecker P, Bookstein FL. 2005. Semilandmarks in three dimensions. In: Slice DE, editor. Modern morphometrics in physical anthropology. Boston (MA): Springer. p. 73–98.

[icab031-B263494637] Hallgrímsson B, Jamniczky H, Young NM, Rolian C, Parsons TE, Boughner JC, Marcucio RS. 2009. Deciphering the palimpsest: studying the relationship between morphological integration and phenotypic covariation. Evol Biol 36:355–76. 23293400 10.1007/s11692-009-9076-5 PMC3537827

[icab031-B21] Hellert SM. (2019). Locomotion transitions and sexual dimorphism: understanding the causes of phenotypic integration patterns [Doctoral dissertation]. Indiana University.

[icab031-B22] Lebrun R, Orliac MJ. 2016. MorphoMuseuM: an online platform for publication and storage of virtual specimens. Paleontol Soc Pap 22:183–95.

[icab031-B23] Machado FA, Hubbe A, Melo D, Porto A, Marroig G. 2019. Measuring the magnitude of morphological integration: the effect of differences in morphometric representations and the inclusion of size. Evolution 73:2518–28. 31595985 10.1111/evo.13864 PMC6895406

[icab031-B24] Olson EC, Miller RL. 1958. Morphological integration. Chicago: University of Chicago Press.

[icab031-B25] Pavlicev M, Cheverud JM, Wagner GP. 2009. Measuring morphological integration using eigenvalue variance. Evol Biol 36:157–70.

[icab031-B26] Pyron RA, Burbrink FT. 2014. Early origin of viviparity and multiple reversions to oviparity in squamate reptiles. Ecol Lett 17:13–21. 23953272 10.1111/ele.12168

[icab031-B27] Randau M, Goswami A. 2018. Shape covariation (or the lack thereof) between vertebrae and other skeletal traits in felids: the whole is not always greater than the sum of parts. Evol Biol 45:196–210. 29755151 10.1007/s11692-017-9443-6 PMC5938317

[icab031-B28] Rhoda D, Polly PD, Raxworthy C, Segall M. 2021. Morphological integration and modularity in the hyperkinetic feeding system of aquatic‐foraging snakes. Evolution 75:56–72. 33226114 10.1111/evo.14130

[icab031-B29] Ripley B, Venables B, Bates DM, Hornik K, Gebhardt A, Firth D, Ripley MB. 2013. Package ‘mass’. Cran r 538:113–20.

[icab031-B30] Rohlf FJ, Slice D. 1990. Extensions of the Procrustes method for the optimal superimposition of landmarks. Syst Biol 39:40–59.

[icab031-B31] Rohlf FJ, Marcus LF. 1993. A revolution morphometrics. Trends Ecol Evol 8:129–32. 21236128 10.1016/0169-5347(93)90024-J

[icab031-B32] Rohlf FJ, Corti M. 2000. Use of two-block partial least-squares to study covariation in shape. Syst Biol 49:740–53. 12116437 10.1080/106351500750049806

[icab031-B33] Savitzky AH. 1983. Coadapted character complexes among snakes: fossoriality, piscivory, and durophagy. Am Zool 23:397–409.

[icab031-B34] Smouse PE, Long JC, Sokal RR. 1986. Multiple regression and correlation extensions of the Mantel test of matrix correspondence. Syst Zool 35:627–32.

[icab031-B35] Vidal-García M, Keogh JS. 2017. Phylogenetic conservatism in skulls and evolutionary lability in limbs–morphological evolution across an ancient frog radiation is shaped by diet, locomotion and burrowing. BMC Evol Biol 17:1–15. 28693418 10.1186/s12862-017-0993-0 PMC5504843

[icab031-B36] Vidal‐García M, Bandara L, Keogh JS. 2018. ShapeRotator: an R tool for standardized rigid rotations of articulated three‐dimensional structures with application for geometric morphometrics. Ecol Evol 8:4669–75. 29760906 10.1002/ece3.4018 PMC5938466

[icab031-B37] Wagner GP. 1996. Homologues, natural kinds and the evolution of modularity. Am Zool 36:36–43.

[icab031-B38] Wagner GP, Pavlicev M, Cheverud JM. 2007. The road to modularity. Nat Rev Genet 8:921–31. 18007649 10.1038/nrg2267

[icab031-B39] Zelditch ML, Swiderski DL, Sheets HD. 2004. Geometric morphometrics for biologists: a primer. Cambridge (MA): Academic Press.

