# Supplementary material for: Local Superimpositions Facilitate Morphometric Analysis of Complex Articulating Structures
Source: Integr Comp Biol. 2021 Apr 27;61(5):1892–904. doi: 10.1093/icb/icab031 (PMC8699094; doi:10.1093/icb/icab031)
Supplement: icab031_Supplementary_Data [file icab031_supplementary_data.zip › icb-2021-0092-File012.doc]

**Supplemental Figure 1.** The choice of reference configuration (i.e., the choice of how to position, scale, and orient the landmark subsets) is consequential. PCA morphospaces of the colubriform snake dataset from the matched local superimpositions procedure using three different reference configurations. A) the mean shape of a global superimposition. B) *Micrurus lemniscatus*. C) *Erpeton tentaculatum*. Note how the percent of shape variation explained by each PC axis changes slightly between reference configurations.

**Supplemental Figure 2.** Taxa used in the snake and wrasse datasets and their phylogenetic relationships.

**Supplemental Figure 3.** Linear regression of PC1 scores of the global and matched local superimpositions from the snake dataset.

**Supplemental Figure 4.** Linear regression of PC2 scores of the global and matched local superimpositions from the snake dataset.

**Supplemental Figure 5.** Linear regression of PC1 scores of the global and matched local superimpositions from the wrasse dataset.

**Supplemental Figure 6.** Linear regression of PC2 scores of the global and matched local superimpositions from the wrasse dataset.

**Supplemental Figure 7.** PCA morphospace of the wrasse dataset after a global superimposition.

**Supplemental Figure 8.** PCA morphospace of the wrasse dataset after matched local superimpositions.

**Supplemental Figure 9.** PCA morphospace of the snake dataset after a global superimposition.

**Supplemental Figure 10.** PCA morphospace of the snake dataset after matched local superimpositions.

**Supplemental Figure 11.** Procrustes distance matrix of the wrasse dataset after a global superimposition.

**Supplemental Figure 12.** Procrustes distance matrix of the wrasse dataset after matched local superimpositions.

**Supplemental Figure 13.** Procrustes distance matrix of the snake dataset after a global superimposition.

**Supplemental Figure 14.** Procrustes distance matrix of the snake dataset after matched local superimpositions.
