# Supplementary material for: Local Superimpositions Facilitate Morphometric Analysis of Complex Articulating Structures
Source: Integr Comp Biol. 2021 Apr 27;61(5):1892–904. doi: 10.1093/icb/icab031 (PMC8699094; doi:10.1093/icb/icab031)
Supplement: icab031_Supplementary_Data [file icab031_supplementary_data.zip › icb-2021-0092-File013.doc]

library(Morpho)

library(geomorph)

\# helper functions

\# centroid1 calculates the centroid position of a landmark configuration

centroid1 <- function(lm){apply(lm, 2, mean)}

\# translate.lm translates a matrix or all the lm configurations in array to a common position

\# 'coords' is a 3D coordinate array

\# 'centroid' is the xyz coordinate you want to translate to

translate.lm <- function(coords,centroid = c(0,0,0)){

\# 'trans1' is from 'Morphometrics with R' by Julien Claude, translates lm configuration to origin

trans1 <- function(M) {M-matrix(centroid1(M),nrow(M),ncol(M),byrow=T)}

k <- dim(coords)[[2]]

if(is.matrix(coords)){

A<-matrix(NA,nrow=nrow(coords),ncol=ncol(coords))

coords <- trans1(coords)

A[,1]<-coords[,1]+centroid[1]

A[,2]<-coords[,2]+centroid[2]

if(k==3){A[,3]<-coords[,3]+centroid[3]}

dimnames(A)<-dimnames(coords)

return(A)}

else{

A<-array(NA,dim=dim(coords))

for(i in 1:dim(coords)[[3]]){

coords[,,i] <- trans1(coords[,,i])}

A[,1,]<-coords[,1,]+centroid[1]

A[,2,]<-coords[,2,]+centroid[2]

if(k==3){A[,3,]<-coords[,3,]+centroid[3]}

dimnames(A)<-dimnames(coords)

return(A)}

}

\# matchLM

\# scales, translates, and rotates, a landmark configuration to a reference

\# array: a (p x k x n) array of the locally superimposed landmark configurations

\# ref: a (p x k) matrix of the lm of the corresponding bone from the reference configuration

matchLM <- function(array,ref)

{require(Morpho)

\# mat2homg & homg2mat from Morpho source code

mat2homg <- function(x) {

x <- rbind(t(x),1)

return(x)}

homg2mat <- function(x) {

m <- nrow(x)

x <- t(x[1:(m-1),])

return(x)}

p <- dim(array)[1]

k <- dim(array)[2]

n <- dim(array)[3]

M <- array

if(dim(ref)[[2]]==3){ref.centroid <- c(mean(ref[,1]),mean(ref[,2]),mean(ref[,3]))}

else{ref.centroid <- c(mean(ref[,1]),mean(ref[,2]))}

ref.cSize <- cSize(ref)

M.ts <- translate.lm(M*ref.cSize,ref.centroid)

M.ts.rot.all <- array(NA,dim=c(p,k,n))

trafo <- getTrafo4x4(rotonto(x=ref,y=mshape(M.ts)))

for(i in 1:n) {M.ts.rot.all[,,i] <- homg2mat(trafo %*% mat2homg(M.ts[,,i]))}

dimnames(M.ts.rot.all) <- dimnames(array)

return(M.ts.rot.all)

}

\# orp, center, center.scale, and fast.center are geomorph support functions

\## tangent projections

orp<-function(A){

if(is.array(A)) {

dims <- dim(A)

n <- dims[3]; k <- dims[2]; p <- dims[1]

Y <- lapply(1:n, function(j) A[,,j])

} else

if(is.list(A)){

Y <- A

n <- length(A); dims <- dim(A[[1]]); k <- dims[2]; p <- dims[1]

} else stop("Input must be either a list or array")

Y1 <- as.vector(center.scale((Reduce("+", Y)/n))$coords)

oo <- matrix(1,n)%*%Y1

mat <- t(matrix(unlist(Y),k*p,n))

Xp <- (mat%*%(diag(1,p*k) - (tcrossprod(Y1)))) +oo

lapply(1:n, function(j) matrix(Xp[j,],p,k))

}

\# center.scale

\# center and divide matrices by centroid size; faster than scale()

\# used in other functions for gpagen

center.scale <- function(x) {

x <- center(x)

cs <- sqrt(sum(x^2))

y <- x/cs

list(coords=y, CS=cs)

}

\# center

\# centers a matrix faster than scale()

\# used in various functions where mean-centering is required

center <- function(x){

if(is.vector(x)) x - mean(x) else {

x <- as.matrix(x)

dims <- dim(x)

fast.center(x, dims[1], dims[2])

}

}

fast.center <- function(x, n, p){

m <- colMeans(x)

x - rep.int(m, rep_len(n, p))

}

\# local.gpa

\# locally superimposes each defined subset of landmark coordinates,

\# scales, rotates, and translates them to the relative size, orientation, and position of the corresponding landmarks in the reference configuration

\# input:

\# coords = array of the raw landmark data

\# partition = index of which 'module' each landmark belongs to, in same order as coords

\# ref = NULL = matrix of landmark coordinates to be used as the reference (i.e., a single specimen), if NULL the mean shape of coords is used

\# *** slide semi-landmarks BEFORE local.gpa ***

\# no tangent projections until all the locally-superimposed coordinates are combined

local.gpa <- function(coords, partition, ref = NULL){

trans1 <- function(M) {M-matrix(centroid1(M),nrow(M),ncol(M),byrow=T)}

if(is.null(ref)){ref <- mshape(gpagen(coords, print.progress = FALSE)$coords)}

else{ref <- trans1(ref)}

ref <- ref*(1/cSize(ref))

u <- unique(partition)

l <- length(u)

lsc <- c()

for(i in 1:l){

proc <- gpagen(coords[which(partition==u[[i]]),,], print.progress = FALSE, Proj = FALSE)

A <- proc$coords

lsc[[i]] <- matchLM(array = A, ref = ref[which(partition==u[[i]]),])

}

out1 <- bindArr(lsc)

out2 <- orp(out1)

out <- simplify2array(out2)

dimnames(out) <- dimnames(coords)

return(out)

}

\# locally superimposing the snake data

lm <- readland.tps(file="snake_landmarks.tps",specID="ID")

\# make the 'partition', indices of which 'module' each landmark belongs to

lgpa.partition <- c(

dentary.p <- seq(1,1,length.out=180),

compound.p <- seq(2,2,length.out=188),

quadrate.p <- seq(3,3,length.out=270),

supratemporal.p <- seq(4,4,length.out=93),

pteyrgoid.p <- seq(5,5,length.out=154),

ectopteyrgoid.p <- seq(6,6,length.out=111),

palatine.p <- seq(7,7,length.out=112),

maxilla.p <- seq(8,8,length.out=227))

\# locally superimposing the bones

\# onto the mean shape (default)

test <- local.gpa(coords = lm, partition = lgpa.partition)

#spheres3d(mshape(test),radius=0.001)

#checkLM(test)

\# anchor onto M. lemniscatus

test2 <- local.gpa(coords = lm, partition = lgpa.partition, ref = lm[,,10])

#spheres3d(mshape(test2),radius=0.001)

\# anchor onto E. tentaculatum

test3 <- local.gpa(coords = lm, partition = lgpa.partition, ref = lm[,,2])

#spheres3d(mshape(test3),radius=0.001)
